# Notes on Chloroform
*Abstract of a paper read before the Baltimore Pathological Society, on May 6th 1870.


**Published:** 1870-07

**Authors:** M. J. DeRosset

**Affiliations:** Professor of Chemistry, Baltimore College of Dental Surgery.


### AMERICAN JOURNAL OF DENTAL SCIENCE.

Vol. IV. THIRD SERIES--JULY, 1870. No. 3.

### ARTICLE I.

### *Notes on Chloroform.**

By M. J. DeRosset, M. D.

Professor of Chemistry, Baltimore College of Dental Surgery.

It is a matter of regret that so little is known, while so much is surmised, concerning the method by which the peculiar physiological phenomena of this agent are produced; and yet, if we bear out of consideration the obvious chemical and mechanical actions of a certain limited number of substances, the same remark is applicable to almost every member of the materia medica, quoad their relation to physiological processes.

Nevertheless the instantaneous manifest, and marvelous changes which accompany the inhalation of $CHCl_3$, no less than the ineffable benefits and it may be deplorable dangers, which ensue therefrom, seem to call for a more special investigation.

Hypothesis has done more than its share in this respect, while experimentation has been too much neglected.

Abstract reasoning upon this subject, analogical or other can not be relied upon to furnish data which experience may

*Abstract of a paper read before the Baltimore Pathological Society, on May 6th 1870.

not refute—while there is nowhere presented a better opportunity for the discovery of truth than is offered by patient and skillful experiment with this agent.

Some things are, however, known to us as facts, and others wear so markedly the facies of facts that we are compelled to accept them as such, and these I propose to present to this Society in the form of *Theses*.

1st. Local anaesthesia from chloroform is limited to those nerves and proportions thereof with which the agent comes into direct contact; but if the trunk of a nerve is brought under its influence the anæsthesia will be more wide spread towards the peripheral end.

2d. General anæthesia is produced only by direct contact of the agent with the ganglionic centres in which it effects chemical or mechanical changes of structure.

3d. It is conveyed to the nerve centres through the medium of the vascular system—and produces no chemical change on the liquor sanguinis or the discs.

4th. The operation is first exhibited upon the cerebral hemispheres, as is evinced by the impairment of the intellectual faculties, (reason, judgement memory) and upon the cerebellum, as is shown by the absence of co-ordination, and finally upon the centres of excitability (motor).

4th. The nature of this operation is unknown, but it may have several factors—diminished histogenesis and histolysis, solution of fatty nerve elements and of protogon, resulting in alteration or suspension of function.

6th. Sudden deaths in surgical practice have not become more frequent since the introduction of chloroform.

7th. Death by asphyxia, with its usual phemonena, is extremely rare; but it may happen if by a too rapid administration, atmospheric air is excluded; it may also happen from paralysis of hypoglossal and motor filaments of glosso-pharyngeal (permitting the tongue to fall back and obstruct the glottis), and of inferior branch of vagus (relaxing the vocal cords).

But death from asphyxia proper rarely and perhaps never occurs from any cause except the exclusion of air.

8th. Chloroform death, in almost every case, proceeds from syncope, the heart being paralysed.

Local anæsthesia of the periphery of the sensative filaments of the vagus and sympathetic nerves in the lungs and heart would prevent the reception of impressions for conveyance to the ganglionic centres; hence there would be no reflex-motor action to the heart and lungs, and the death would be from an eccentric cause. Or the ganglia form which the vagus derives its motor filaments (hypoglossal, facial and spinal accessory) may be overpowered by immediate contact with the agent causing the arrest of the heart's movements —the cause in this instance being centric.

9th. The too rapid administration may supersaturate the blood. Chloroform is very slightly soluble in water or blood —a slow cautious inhalation may introduce no more than can be readily taken off into the circulation in a state of solution—but if rapidly introduced there may happen a collection of gaseous chloroform undissolved, (and perhaps even separated in a liquid form), producing a spumous, frothy state of the blood, from which death would ensue with symptoms similar to those which follow the introduction of air into the vessels. And indeed the phenomena of death from chloroform simulate, generally in every essential particular, those which are described as the consequence of this accident.

The cause of death from the introduction of air is as yet not determined, but it is easy to understand how frothy bloody or heavy liquid chloroform passing through the minute vessels of the medulla or other portion of the cerebro-spinal axis would determine the immediate abrogation of its functions.

In presenting these theses and the hypothesis which forms the last one, it is proper to state that they have not been deduced from a consideration of the superficial analogies which $CHCl_3$ may bear to other anæsthetic agents, for indeed less is known of these probably than of it, and analogical methods of reasoning yield generally fallacies unless they start from fixed data. It is manifest however that the less or greater number of chlorine equivelants in a given substance can not be regarded as determining its anæsthetizing power since a great many are found possessed of the same property, which contain no atom of this element, conspicuously, $N_2$ O; $C_4$ &$_{10}$ O; $C_5$ $H_{11}$, $NO_2$ this latter being the most promising agent yet discovered.

In insisting upon the extreme variety of chloroform death by primary asphyxia, I am not unmindful of the striking experiments conducted by a celebrated veterinary surgeon in France and published in 1867, in the Archives Generales, but, the phenomena usually attending the death of a human being while inhaling chloroform are so entirely different from those exhibited in cases of death by apnœa from other causes, that the conclusion is inevitable.

It should always be administered slowly and gradually, which is contrary to the opinion urged by some authorities, but which I consider sufficiently established by the facts.

The amount of chloroform in the inspired air should not exceed 1 or 2 per cent. or less than one half cu. inch of gaseous chloroform ; it diffuses less rapidly than air, in the ratio of about 1 to 18. An elevated temperature determines, ceteris paribus, its more rapid conversion into vapor as does a low state of the barometer.

The best inhaler is a cambric handkerchief folded twice, upon which ten or twenty minims may be poured after every ten or twelve inspirations. Chisolm's inhaler is the most valuable of all the apparatus especially designed for the purpose—and it has the advantage of economy over the simple method. All other inhalers for chlorform are worse than useless.

In cases of threatened death, artificial respiration should be instantly resorted to with a view of moving the current of blood through the lungs —it should be thoroughly performed, about 30 times a minute—the directions, hitherto observed, to imitate the natural movements being faulty in principle, and not adequate to supply the demands of an embarrassed circulation.

The larynx and trachea should be kept open by whatever necessary means, even to the extent of incision into the latter, and Dr. Richardson's bellows may be tried, or the ordinary hand bellows.

The advantage of a postural treatment, on the left side, as adopted at the London ophthalmic hospital, is not borne out by any scientific considerations and experience has as yet been too limited to justify its adoption in inconvenient cases to the exclusion of more promising methods.

The application of a mild interrupted current of electricity to the phrenic sympathetic vagus nerve should never be neglected. The current should pass from the cardiac and epigastric regions towards the superior portion of spinal cord and medulla oblongata, the positive electrode being placed anteriorly and the negative one on the nape of the neck.

Singular and indiscriminate application of electricity is pernicious.

The object of electricity is to supply artificially the stimulus which the besoin de respirer normally gives to the lungs, and the excito-motor action which the passage of blood through the the heart produces. The reflex movement thus induced through the motor fibres of vagus and phrenic determines the movement of these organs. Furthermore, the artificial galvanisation of the sympathetic in the neck determines a more powerful contraction of the peripheral vessels of the brain and other parts, pouring onwards their contents, whether these be blood, frothy blood or liquified chloroform, as assumed in the 9th thesis.

In desperate cases the injection into the veins of diluted ammonia water may be tried to arouse the sinking nerve force, and to contribute towards the preservation of the fluidity of the blood.

A similar use of a solution strychnia, may be tried, in as much as this alkaloid given hypodermically in cases of impending death from chloral has promptly alleviated the symptoms, moreover chloroform is our readiest means for overcoming the tetanic symptoms of strychnia.

---

### ARTICLE II.

### *Prophylaxis or Prevention of Dental Decay.*

